# Understanding abortion seeking care outside of formal health care settings in Cape Town, South Africa: a qualitative study

**DOI:** 10.1186/s12978-021-01243-3

**Published:** 2021-09-23

**Authors:** Jane Harries, Kristen Daskilewicz, Tshegofatso Bessenaar, Caitlin Gerdts

**Affiliations:** 1 Women’s Health Research Unit, School of Public Health and Family Medicine, University of Cape Town, Cape Town, South Africa; 2 Ibis Reproductive Health, Johannesburg, South Africa; 3 Ibis Reproductive Health, 1736 Franklin St, Oakland, CA USA

**Keywords:** Abortion, Decision-making, Out of clinic abortions, Informal sector abortions, Qualitative research, South Africa

## Abstract

**Background:**

Although abortion was legalized in South Africa in 1996, barriers to safe, legal abortion services remain, and women continue to seek abortions outside of the formal healthcare sector. This study explored the decision-making processes that women undertake when faced with an unintended pregnancy, the sources of information used to make their decisions and the factors that contribute to their seeking of informal sector abortion in Cape Town, South Africa.

**Methods:**

We conducted 15 semi-structured in-depth interviews in English with women who had accessed an abortion outside of the formal health care sector. Women were recruited with the assistance of a community-based key informant. Data was analyzed using a thematic analysis approach.

**Results:**

Participants were aware that abortions were legal and accessible in public clinics, however they were concerned that others would find out about their unintended pregnancy and abortion if they went to legal providers. Women were also concerned about judgment and mistreatment from providers during their care. Rather than seek care in the formal sector, women looked past concerns around the safety and effectiveness of informal sector abortions and often relied on their social networks for referrals to informal providers.

**Conclusions:**

The findings highlight the decision-making processes employed by women when seeking abortion services in a setting where abortion is legal and demonstrate the role of institutional and societal barriers to safe abortion access. Abortion service delivery models should adapt to women’s needs to enhance the preferences and priorities of those seeking abortion care-including those who prefer facility-based care as well as those who might prefer self-managed medical abortions.

## Plain language summary

Although abortion was legalized in South Africa in 1996, barriers to safe, legal abortion services remain, and women continue to seek abortions outside of the formal healthcare sector. This study explored the decision-making processes that women undertake when faced with an unintended pregnancy, who they discuss and seek help from, and the factors that influence their decision to seek an abortion outside of the formal health care sector. We interviewed 15 women who had obtained an abortion outside of the health care sector in Cape Town, South Africa. Women were aware that abortions were legal and available in public clinics, but they were concerned about negative and judgmental attitudes from health care providers even though some women were aware of the possible health safety issues related to seeking an abortion outside of the clinic setting. Abortion services should adapt to women’s needs and offer them options of facility-based care but also self-managed medical abortions under the guidance of health care providers.

## Background

The South African Choice on Termination of Pregnancy Act of 1996 states that a pregnancy may be terminated at a woman’s request up to 12 weeks gestation. Beyond 12 weeks and up to 20 weeks gestation, an abortion may be performed for any of the following reasons: the pregnancy would pose a risk to the woman’s physical or mental health; there is a substantial risk that the fetus would suffer from severe physical or mental abnormality; the pregnancy resulted from rape or incest, or the continued pregnancy would significantly affect the social and economic circumstances of the woman. From 20 weeks gestation onward, abortions are available under very limited circumstances. Registered nurse midwives who have undergone abortion training are permitted to provide abortions up to 12 weeks gestation. Abortions in the second trimester (13–20 weeks) can only be performed by a doctor. Abortions are provided free of charge in public health care facilities designated to provide abortion care services [1].

Different abortion methods are available in public sector facilities depending on gestational age, availability of trained providers, and availability of different methods across the nine provinces in South Africa. Medical abortion is available using mifepristone and self-administered misoprostol up until 63 days gestation and manual vacuum aspiration up until 12 weeks gestation. Second trimester abortions using the dilatation and evacuation method are available on a limited basis. Labor induction with misoprostol used alone is the current standard of care in South Africa for second trimester abortion [1].

Despite the legal availability of abortion services in South Africa post 1996, barriers to safe and legal abortion access remain. These barriers include provider opposition to rendering or participating in abortion services, often on the grounds of religious or moral beliefs, stigma associated with abortion, a shortage of trained abortion providers, especially second trimester providers, and poor knowledge of the abortion legislation amongst women seeking care [2–5]. As a result of these barriers, a high proportion of abortions in South Africa take place outside of the formal health care sector [6–10]. For the purposes of this study, we use the term ‘informal abortion’ to refer to induced abortions that occur outside of the formal health care sector (outside clinic abortions) without medical assistance either through a self-managed abortion or through accessing an unlicensed provider providing medication or other substances to induce an abortion.

While we know that women in South Africa continue to seek abortions outside of clinic settings, research has, to date, not been undertaken to explicitly explore and document women’s decision-making processes to seek care from informal sector providers or to self-manage abortions. Existing research documenting women’s experiences with terminating their pregnancies in the informal sector in South Africa relies on the recruitment of women from formal healthcare facilities, representing women who were able to access the formal health care system at some point during the process of abortion seeking [7–9], and thus may be excluding the perspectives of those who face the greatest barriers to care. A study in Cape Town, South Africa on women’s experiences with informal sector abortions that recruited women using peer-to-peer recruitment found most women did not interact with the formal healthcare setting at any point during their informal sector abortion process [7], highlighting the need for research centered on the experiences of women who never reach formal healthcare services in order to understand the complex factors that impact women’s trajectories to abortion related care [10].

Little is known about the decision-making process and reasons why women seek informal abortions in settings where abortion is legally available [11]. This study aims to explore the decision-making processes that women undertake when faced with an unintended pregnancy, the sources of information used to make their decisions and the factors that contribute to their seeking of informal sector abortion in Cape Town, South Africa. Results from this study can help inform public health interventions, policy, and advocacy aimed at improving and expanding access to safe and legal abortion, quality of care and support, and potentially averting abortion related morbidity and mortality.

## Methods

### Study population and recruitment

The study was conducted in Cape Town, South Africa between February to June 2017. Recruiting women who had accessed informal abortions proved to be difficult. We initially tried recruiting women through social media and informational flyers placed at sexual and reproductive health non-government organizations (NGOs) inviting potential respondents to participate in a research study on outside of a clinic or self-induced abortion experiences. Despite these efforts, no one responded to the requests for study participation via NGO contacts, flyers, or social media. We subsequently decided to use a community-based key informant to assist in recruiting study participants through community networks. Study participants were recruited via a key informant who was previously involved in one of our prior studies exploring women’s experiences seeking informal abortion services [7]. Participants were eligible to participate in the study if they attempted to end a pregnancy outside of the formal health sector in the past five years, were aged 18 years or older and were able to provide informed consent. We use the term “women” whilst acknowledging that not all abortion seekers are women. However, our participants self-identified as women and we thus use the language as it applied to the study population.

### Data collection

Two female research assistants trained in qualitative research methods with prior experience in abortion related research, followed up telephonically with contact details provided by the key informant and set up a time and place to meet potential study participants. Interviews were conducted face to face in a private space and were 45–60 minutes duration. All interviews were conducted in English and audio recorded with participant consent. Recordings were transcribed verbatim by an independent transcriber. A total of 15 participants were interviewed.

The research instrument in the form of an interview guide was open-ended and included probes for potential additional issues that could emerge as important concerns. The interview guide was pilot tested. Field reflective notes were recorded after each interview, which provided additional context when reviewing the transcripts.

### Data analysis

Data were analysed using a thematic analysis approach, in which main themes and categories were identified and analysed within and across data. Initial categories for analysing data were drawn from the interview guide and key research questions, and then themes and patterns were identified after reviewing the data.

Data analysis began by reading transcripts, creating memos, and assigning initial codes. Using this process, we identified recurring themes and developed further emergent codes to complement initial deductive codes, which were derived from the research questions and interview guide. A code book was developed by the first author (JH) and the transcripts were coded by the Principal Investigator (JH) and a research assistant (KD) who conducted the interviews. Following initial analysis and codebook finalization, interviews were entered into the qualitative software package NVivo 12 Pro which facilitated the sorting and management of the data Coded transcripts were cross checked by co-author (KD) for coder variation. The codes developed were deductive in the first instance with initial codes and definitions developed based on key domains of enquiry which included: reasons for seeking an abortion outside of the formal sector, abortion experiences and outcomes and the influence of social networks in the abortion seeking trajectory. Additional inductive codes were added during the coding process, as expected or unanticipated issues were identified. These included the importance of social networks (close friends) in assisting women accessing an informal abortion. Memos were recorded alongside the coding process and were useful in exploring relationships of links across categories, or reflections about a particular phenomenon [12]. The data was then reviewed for major trends, crosscutting themes were identified, and issues for further exploration were prioritized for final analysis.

Study rigor was maintained throughout the study. Dependability was addressed by providing a detailed discussion of the study methods. We established an audit trail by developing a detailed track record of the data collection process, coding was undertaken by more than one person and checked amongst the study team for coder variation. Coding discrepancies were resolved through discussion and consensus. Confirmability was addressed, all interviewers kept a reflective journal and we held regular researcher team meetings to discuss research progress. Data saturation was achieved.

Participant quotations are presented to illustrate the themes and findings from the analysis. The findings of the study have been reported following the Consolidated Criteria for Reporting Qualitative Research (COREQ).

### Ethical considerations

Ethical approval to conduct the study was obtained from the Human Research Ethics Committee, University of Cape Town. All study participants provided written informed consent prior to the interview process. Verbal permission was obtained prior to digitally recording all interviews. Confidentiality and anonymity were ensured. Participants were assured that in all forms of dissemination, including publications and dissemination meetings, participants would not be identified by name or any other identifier. All data were closely controlled and stored in locked files and password protected computer files. Digital recordings were erased once they had been cross checked after data transcription. All women were reimbursed ZAR (South African Rand) 100 for their time and to cover transport.

## Results

Socio-demographic characteristics were collected from all 15 study participants. The mean age was 35.2 years (range 26–45), 80% (12/15) were employed with just almost half (7/15) involved in sex work, all but four participants had children, and none were married. Of all participants, thirteen had at least one informal sector abortion and two had accessed two informal abortions. Eleven of the participants had purchased and used herbal mixtures including aloe vera, traditional remedies and purgatives to terminate their pregnancy. Herbal and traditional mixtures were taken orally and often mixed with concentrated orange juice, milk, or alcohol (vodka). Herbal mixtures including aloe vera were purchased from traditional healers whereas traditional remedies and other products including purgatives, were purchased from chemists. Four of the participants had accessed a non-licensed provider and took unknown tablets that induced bleeding assumed to be misoprostol.

Key themes for exploring complex decision-making in seeking an informal abortion included (i) social networks and other sources of information in the abortion seeking trajectory; (ii) the method of abortion and reported outcome including seeking post abortion care and (iii) reasons for seeking an informal abortion.

Overall, complex decision-making informed by individual and personal circumstances underscored by reticence towards the formal healthcare sector motivated seeking an abortion in the informal sector. Rather than seeking care within the formal health sector most women relied on social networks for referrals and endorsements to an informal provider or traditional methods to induce an abortion. However, five women did access the formal health care sector for post- abortion care.

## Sources of informal abortion information

### Social networks

Social networks played an important role in women’s abortion seeking trajectory and in supporting and advising women how to access an informal abortion. Upon considering themselves pregnant, most women disclosed and sought advice about informal abortion options from friends who had personal experience accessing informal abortions, knew someone who could perform the informal abortion or knew how to self-induce using a self-prepared herbal mixture or substance.

Advice and assistance in obtaining an informal abortion was further underscored by friends vouching for the safety of a provider or particular method. Women discussed how knowing a friend who had successfully had an informal abortion without health complications influenced their decision to opt for an informal abortion.

A participant recounted how her friend endorsed an unlicensed provider based on his perceived safety. The unlicensed provider provided her with two white tablets.

*She [friend] also had the same abortion … like 10 or so months before and I knew about it that was why I spoke to her. And then she showed me okay, it’s this doctor, she showed me like in the paper, he’s got a regular ad in there and the same doctor’s also got stickers all over in the train, … so she just showed me that’s the one that she used. The reason why she encouraged me to use him because when she went there, compared to the other places that she’s heard of or the other stories, his place was clean … I trust her and somehow it made me feel a little bit better knowing that one of my friends went to the person.*

Another participant discussed her need for an abortion with a trusted friend who in turn suggested a traditional herbal medicine and offered to help in sourcing the herbal medicines.

*I bought some things, there was a friend of mine she had an abortion also, so she told me what to do what to *buy*…and then she escorted me to go and get them…”*

Participants sources of information extended beyond their social networks to include the proliferation of advertisements for “safe and quick abortions” in public spaces (trains and community newspapers) which were often endorsed by friends. These unlicensed providers all provided unknown oral tablets to induce abortion presumably misoprostol.

*“I saw an advert and then I spoke to a friend who had also been to the same person, they call him, like a herbalist … he had an advert in the paper and also on the train, these stickers that they give out and it said there are abortions for R 300 like in 30 minutes…”*

### Method of abortion

Participants discussed employing three different methods when attempting an informal abortion. These included: drinking a mixture prepared by a traditional healer; contacting a non-licensed provider who provided oral abortifacients and preparing or buying a mixture for a self -managed abortion. Trusted social networks again played a crucial role in guiding women in accessing these methods.

### Traditional healer

Some participants described utilizing the services of a traditional healer recommended by friends who had previously accessed their services for an informal abortion. Participants who utilized the services of a traditional healer mostly described the abortion mixture as brown or white in colour, having an offensive taste and in some cases using dried aloe vera.

*I go to traditional doctor near the taxi rank to give me some, aloe vera…I drink that aloe vera. it tastes horrible…I take only a spoon because it is too much strong…if you take the three spoons you are going to die that guy [traditional healer] tells you…*

Some participants however discussed paying a traditional healer for a list of reportedly easily obtainable herbal and other ingredients and received instructions on how to prepare the mixture themselves.

### Unlicensed providers

Unlicensed providers who advertised their services illegally online, on trains and other public spaces most commonly supplied participants with unmarked tablets of varying sizes, shapes, and colour to terminate their pregnancy. Some participants were instructed to swallow the tablets at specified times and to expect vaginal bleeding.

*He gave me two pills and told me I must take one when I leave his office. The second one* [*tablet*]*, I must take it when I get home…it was two of the same pills to me it looked the same, I didn’t see any name on the tablet …He just said to me it will basically be like getting period pains and *then* the bleeding will come down. He said most of the bleeding should happen in a space of 24 hours…it should be complete and then just like normal periods, they can last up to 2 weeks …*

### Self-abortion mixture

All participants who attempted an informal abortion using a self-abortion mixture were informed of the method by a friend who had successfully used the mixture to induce an abortion.

A friend of mind told me how to do an abortion because she had one and she did it the same way…She told me how to prepare the mixture…and we made it at my friend’s place.

### Reported outcomes

Some participants reportedly sought formal medical intervention after attempting an informal abortion. Reasons for doing so included experiencing complications such as a possible infection or bleeding or when they considered the informal abortion to be unsuccessful.

### Reported infection

*After [attempting informal abortion], I developed an intense fever and I said okay I’m going to go to the clinic just to check it out and to tell them what happened… I went there and I spoke to the Sister [nurse] and I told her everything and she shouted at me, told me how stupid I was, why didn’t I come there. I couldn’t tell her exactly why… She [nurse] checked and I ended up *having* an infection., so they treated that at the clinic … and then after that the bleeding stopped and it was better.*

### Reported bleeding

Almost all women (13/15) who attempted an informal abortion whether through tablets or herbal mixtures reported bleeding post abortion. The duration of bleeding ranged from one day to three weeks. Whilst post abortion bleeding is common, women were not fully aware of what to expect, became concerned and a few sought assistance at health care facilities. A participant was concerned about bleeding for three weeks.

*I was still on blood [three weeks after], the blood never stopped…Then I went to the clinic they gave me the *tablets* to stop me [bleeding] because the blood smelt bad, not like normal blood.*

Some participants reported successfully terminating their pregnancy using a mixture of herbs and in one case unknown “white tablets” assumed to be misoprostol.

*Yes, it [white tablets with *concentrated* orange juice] works because… After three days… I went to school, but I was still bleeding that time but not so heavy like before.”*

*I’m worried because it *was* the first time, I’m making abortion, but that method [herbal mixtures and vodka] make me nice… Yes, it works.*

However, a few participants cautioned against accessing illegal (“backyarder”) providers or using dangerous household cleaning materials even though they had not personally attempted these methods.

*What I understand now – go and do a safe abortion to the professionals. Don’t go to the backyarders. Those oro crushes, steel wool, those things, you may end up getting cancer. Instead of going to backyarders go to the professionals.*

Whilst women reported diverse outcomes post abortion most felt that certain methods especially if endorsed by friends were still preferable to accessing the formal health care sector where provider attitudes and treatment were unpredictable.

### Reasons for seeking an informal abortion

Pathways to seeking an informal abortion for many women, especially those who were involved in work in the informal sector, such as sex work, were informed by social stigma. Fear of judgement and mistreatment by clinic staff or privacy and confidentiality concerns were reported by most women as a major consideration influencing their decision to opt for an informal abortion.

A sex worker explained her reasons for seeking an informal abortion were linked to fear of recognition and judgemental attitudes towards sex workers, further underscored by seeking an abortion. Accessing an informal, “backdoor” provider ensured more privacy and anonymity and privacy outweighed safety issues. In sum, she felt being judged as a sex worker and seeking an abortion.

*I don’t like going there [the clinic] because some of the staff, they are familiar with certain female faces, since you are living in the area … And when I go to the clinic then you can feel there’s gossip *about* you … that makes a person feel very uneasy it’s like judging you. You find it difficult to go to the clinics because of how the staff then treats you according to what they know what you do for a living.*

She further explained her reasoning for accessing an informal provider:

*…then that’s when I had no choice … I’m too embarrassed and shy and ashamed to go to the hospital to do it the right way, I’m going to do it the backdoor…. at least no-one’s going to know, less people that see me the better, it’s safer for me.*

Other reasons reported by women for accessing an informal abortion were avoiding delays, especially related to gestational age screening. This influenced their decision to have an informal abortion.

*Sometimes you feel like you take the short way…Sometimes at the clinic you have to go to the scans and those paperwork –it’s a long process. But this one [informal abortion] you know you will do quickly. I know it’s very risky but sometimes when you are desperate you want a short cut.*

However, whilst women accessed the informal sector to terminate a pregnancy,

many participants discussed their preference for allopathic medicine if they or their family required medical attention for minor illnesses such as flu and for chronic medication again reinforcing reluctance to access the formal sector for an abortion due to social stigma and associated attitudes towards seeking an abortion.

*If you got a flu you go to the doctor to get some medication, Disprin* [aspirin] *and Panado* [Paracetamol]*.*

Similarly, a participant accessing the public sector for antiretroviral HIV treatment explained:

*I normally *attend* a public health care facility when I require medical assistance. I do because I am on ARVS, I am confident with my doctor, I know my doctor, I can tell him anything.*

## Discussion

This is one of the few studies conducted in South Africa exploring the decision-making process and reasons why women seek informal abortions in a setting where abortion is free and legally available**.**

Overall, while participants were generally aware of legal provision of abortion in the public sector, they expressed concerns about the quality of care they would receive, with many participants voicing concerns around the lack of privacy and provider mistreatment in public sector services. Fear of mistreatment and social stigma was further enhanced for those participants involved in sex work.

In accounts of decision-making about how to terminate a pregnancy, participants described fears about the perceived physical health risks associated with informal sector abortion prior to their decision to seek care. While we cannot verify the risks of some of the medications taken, fears over perceived medical safety were outweighed by reservations about abortions in the formal health sector, which is similar to research in other countries where abortions are legal [11, 13]. This is not surprising considering the contested nature of abortion globally and the social stigma associated with abortion including in countries where abortion is available on broad grounds [7, 14, 15]. Concerns with abortion services in the public sector highlights the need for non-judgemental, patient- centred abortion care and the importance of expanding self-managed medication abortion in a supportive and enabling environment.

Social networks through trusted friends played an important role in shaping women’s journey to secure an abortion outside of the formal health system. This is not unusual given the personal nature of an unintended pregnancy and decision to terminate a pregnancy. Our findings were similar to studies in other settings where fear of abortion stigma, preference for privacy and support from social networks about informal abortion methods were motivating factors in accessing an abortion outside of the formal health sector [16, 17].

Women's sources of information extended beyond their social networks to include advertising in public spaces. The proliferation of unlicensed providers advertising in public spaces, online and in community newspapers remains a concern and suggests that unlicensed providers remain in demand [8]. The proliferation of “unsafe and illegal providers” is currently part of a Marie Stopes South Africa campaign to shut down and report all illegal and unsafe abortion providers in South Africa [18].

While not all informal methods led to complications and we interviewed women who had not experienced serious long-term complications, the ingestion of household products could lead to serious health conditions. The ethnopharmacology of traditional and herbal medicines reported would be important to explore. Aloe used as an abortifacient mentioned by study participants has been reported in South Africa [19], however it is unknown what the long-term effects of these reported compounds or herbal mixtures are. The combination of herbal and other substances including unknown medical abortion tablets has been reported elsewhere, including the US, where women appear to access a hybrid of more traditional and Western biomedical medicines [16].

The desire for privacy and confidentiality in this study supports an increasing body of evidence documenting people’s preferences in abortion care, and that different models of out of clinic care are safe, acceptable, and sometimes preferable for abortion seekers [20–22]. This aligns with the World Health Organization Guidelines on Self-care Interventions for Health [23] which confirms that self-managed abortion is medically safe if pregnant individuals have information on effective protocols and access to follow up health care if needed [23].

Whilst our study predated the global COVID-19 pandemic, impacts at the health system level and disruptions in regular provision of sexual and reproductive health care services, such as safe abortion and contraceptive services [24] could lead more people to seek out-of-clinic abortions during lockdowns or travel restrictions.

Quarantine and lockdown restrictions during the COVID -19 pandemic can also lead to increased domestic violence especially pertinent in a country like South Africa with one of the highest levels of gender-based violence [24, 25]. This in turn may lead to increased unintended and unwanted pregnancies due to difficulties in accessing contraceptive supplies, increased incidence of domestic violence, and rising income insecurity [26].

## Limitations

There are several limitations to this study. Women recruited for this study were asked to share personal details of their out of clinic abortion experiences. It is likely that there is a difference in women who felt comfortable enough to participate in this study compared to those who chose not to share their personal experiences. Our recruitment through a community partner suggests that participants may have had an existing relationship with the NGO, possibly indicating a higher level of social support than others who might access informal sector abortions.

As a qualitative study, our findings are not meant to be generalizable. Rather, these results highlight the complex decision-making process to seek informal sector abortions in the context where abortions are legal, free of charge in the public sector and available on broad grounds.

## Conclusions

Women in South Africa continue to access abortions outside of the formal health care sector for numerous reasons despite available free abortion services. Our study highlighted the complex decision-making processes employed by women when seeking abortion services and the possible reasons for seeking an informal abortion which were related to fears of mistreatment and judgmental attitudes displayed by health care staff. Informal abortion provision afforded women more privacy and confidentiality, although some participants reported experiencing medical complications.

Abortion service delivery models should adapt in order to centre the preferences and priorities of those seeking abortion care—including those who prefer clinic-based, or surgical care, as well as those who might prefer self-managed approaches. In order to expand access to safe, high quality abortion care, we must meet people’s needs for the care they receive. The health care system needs to ensure that services are safe, judgment free, ensure personal privacy and remain person-centred. Further efforts should be directed towards informing women in all communities about the availability of free abortion services in the public health sector and educating them about the risks of unsafe methods of pregnancy termination.

## Data Availability

The datasets used and/or analysed during the current study are available from the corresponding author upon reasonable request.
